# Proteins Potentially Involved in Immune Evasion Strategies in *Sporothrix brasiliensis* Elucidated by Ultra-High-Resolution Mass Spectrometry

**DOI:** 10.1128/mSphere.00514-17

**Published:** 2018-06-13

**Authors:** Luana Rossato, Leandro Ferreira Moreno, Azadeh Jamalian, Benjamin Stielow, Sandro Rogério de Almeida, Sybren de Hoog, Joanna Freeke

**Affiliations:** a Westerdijk Fungal Biodiversity Institute, Utrecht, The Netherlands; b Faculty of Pharmaceutical Sciences, University of São Paulo, São Paulo, Brazil; c Thermo Fisher Scientific, Landsmeer, The Netherlands; Yonsei University

**Keywords:** *Sporothrix brasiliensis*, *Sporothrix schenckii*, genomics, immune evasion, proteomics, virulence factors

## Abstract

Sporothrix brasiliensis is the prevalent agent of a large zoonotic outbreak in Brazil. With the involvement of several thousands of cases, this is the largest cohort of human and animal sporotrichosis on record in the world. Infections are characterized by local cutaneous dissemination in humans without underlying disease. S. brasiliensis has shown a high degree of virulence in a mouse model compared to the remaining *Sporothrix* species, including the ancestral species, Sporothrix schenckii. The present paper investigates a genomic and expressed-proteome comparison of S. brasiliensis to S. schenckii. Using bottom-up proteomics, we found 60 proteins exclusively expressed in S. brasiliensis. No significant genomic differences were found among the genes coding for this protein set. A comparison with literature data identified nine proteins that are known to be involved in virulence and immune evasion in other species, several of which had not yet been reported for the *Sporothrix* species analyzed.

**IMPORTANCE** Sporotrichosis is an important disease in Brazil that is caused by fungi of the genus *Sporothrix* and affects cats and humans. Our work investigated the proteins differentially expressed by S. brasiliensis in order to find out why this species is more virulent and pathogenic than S. schenckii. We verified a set of proteins that may be related to immune escape and that can explain the high virulence.

## Abstract (precis)

Sporotrichosis is an important disease in Brazil that is caused by fungi of the genus *Sporothrix* and affects cats and humans. Our work investigated the proteins differentially expressed by S. brasiliensis in order to find out why this species is more virulent and pathogenic than S. schenckii. We verified a set of proteins that may be related to immune escape and that can explain the high virulence.

## INTRODUCTION

Sporotrichosis is a subcutaneous mycosis with a global distribution (1, 2) caused by several closely related species of the genus *Sporothrix* (3). The prevalent species associated with the disease are Sporothrix schenckii, S. brasiliensis, and S. globosa (4). Sporotrichosis caused by S. schenckii is classically acquired through traumatic inoculation of the fungus into subcutaneous tissues via contaminated plant debris, thorns, or soil and is often associated with gardening and rural labor; S. globosa also originates from plants (5). Occasional transmission via scratches by cat nails has been reported in S. schenckii. This alternative route of transmission proved very successful in the recently emerging species S. brasiliensis. Zoonotic transmission by domestic cats (Felis catus) in Brazil changed the paradigm of the disease (1). Cats mutually disperse the disease with bites and scratches during fights or by contact with nasal secretions (6). Although feline sporotrichosis is known from scattered infections in several countries, the major zoonotic outbreak in Brazil has been registered recently by Sanitary Surveillance—Rio de Janeiro; the number of cases of feline sporotrichosis was found to have increased compared to the previous year, representing 3,253 feline cases (6, 7), and the disease is spreading to other regions (8, 9). In parallel, the number of human cases in this region has increased progressively. Barros et al. (10) described approximately 2,200 human cases of sporotrichosis diagnosed between 1998 and 2009 in Rio de Janeiro, representing the largest cohort of human and animal sporotrichosis on record in the world. S. brasiliensis is nearly exclusively the etiological agent of feline sporotrichosis in Brazil. The fungus was identified in 96.9% of cat samples, with the identifications being proven by isolation of the pathogen from lesions and subsequent molecular characterization. A correlation between cat outbreaks and the prevalence of S. brasiliensis in humans was found in the same geographic area, with Rio de Janeiro as a focus (11).

Clinical manifestations of sporotrichosis are polymorphic. The disease affects the skin and subcutaneous tissues, usually in the form of regional lymphatic spread (12). Fixed cutaneous and lymphocutaneous forms represent more than 80% of clinical pictures globally (13). However, S. brasiliensis in particular is also associated with disseminated cutaneous infection, hypersensitivity reactions, and mucosal cases, while patients with S. schenckii mostly present with infections that are less severe and more localized (14), and S. globosa causes fixed sporotrichosis almost exclusively (15). Pathogenicity and virulence thus differ significantly between species.

Virulence studies in systemic infection models using immunocompetent mice have shown that S. brasiliensis is the most virulent species, followed by S. schenckii, the rare species Sporothrix luriei, and S. globosa, while the phylogenetically remote species Sporothrix mexicana and Sporothrix pallida have low virulence (16, 17). Similar differences were observed in a subcutaneous mouse model, where S. brasiliensis was more virulent than S. schenckii (18). Some studies revealed that virulence could be variable within the species, with isolates displaying high, moderate, or low virulence (19–21). The main established factors required for virulence in these fungi and consequently for evasion of the immune system are the secretion of toxic factors in extracellular vesicles (22–24), adherence, and morphological conversion from mold to yeast. This phenotypic switch is associated with changes in cell wall composition, the presence of antigenic molecules, and expression of virulence traits. In general, dimorphism is known to be essential for virulence in the mammalian host (25) in pathogenic fungi in *Blastomyces*, *Histoplasma*, *Paracoccidioides*, *Coccidioides*, and *Talaromyces*. In *Sporothrix*, efficient transmission of yeast cells may be associated with the more severe forms of the disease, leading to the hypothesis that the yeast form is more virulent than the mycelial form (26). Other than dimorphism and thermotolerance, current knowledge on *Sporothrix* virulence remains limited. General fungal proteins linked to fungal virulence factors and immune system evasion (27) are, for example, calcium binding protein (CBP) and superoxide dismutase (SOD) in Histoplasma capsulatum, glucan synthase in *Coccidioides* sp., α-1,3-glucan and Drk1 (dimorphism-regulating kinase 1) in several fungi, and *Blastomyces* adhesin-1 (BAD-1) in B. dermatitidis (28). Cell wall proteins and glycoconjugates were identified in S. schenckii, and the relevance to host-fungus interaction and stimulation of the host immune system was underlined (29, 30). Virulence- and immune escape-related proteins identified in S. schenckii further include histidine kinase, phosphatidylinositol 3-kinase (31), 70-kDa protein (32), 44-kDa peptide hydrolase, and a 47-kDa enolase which was predicted to be an adhesion (33). The 3-carboxymuconate cyclase 60-kDa protein was found to be related to virulence in S. brasiliensis (34). However, more information on the identity of the proteins involved in biosynthetic pathways is needed. The main biological questions in the present study concerned proteins expressed in *Sporothrix*. Members of this genus have an evolutionary origin in fungi with a plant- and insect-associated lifestyle, shifting to mammal infectivity in the recently evolved “pathogenic clade” that comprises S. schenckii and its clonal offshoot S. brasiliensis (2). Potential virulence- and immune evasion-related proteins were revealed by comparing the genomics and yeast form-expressed proteome of S. brasiliensis to those of S. schenckii. Ultra-high-resolution mass spectrometry (MS) analyses allowed us to identify proteins involved in immune evasion, metabolism, adhesion, and cell surface and biosynthetic processes.

## RESULTS

### Genomics.

**FIG 1:**
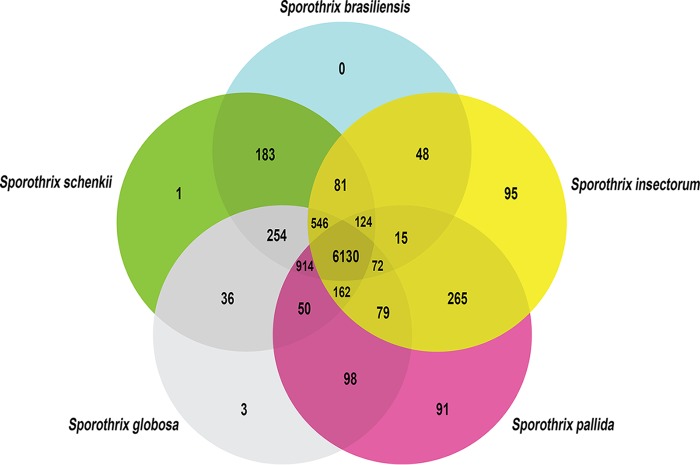
Cluster specifics for S. brasiliensis, S. schenckii, *S. globosa*, S. pallida, and S. insectorum. Data represent the total number of predicted genes in each species, the number of clusters containing orthologs, and the number of species-specific genes.

*In silico* gene prediction was performed by the use of AUGUSTUS (model S. schenckii) for S. pallida and *S. globosa*, resulting in 9,712 and 8,747 predicted genes, respectively. The gene sets of S. brasiliensis, S. schenckii, and S. insectorum were downloaded from GenBank. Orthologous genes were identified using OrthoVenn (35). The total number of predicted genes in each species, the number of clusters containing orthologs, and the number of species-specific genes not assigned to any cluster are shown in Table 1. Overall, we identified the presence of 6,130 clusters of orthologs conserved across the five *Sporothrix* species. None of the clusters were specific for S. brasiliensis. S. schenckii and S. globosa possess 1 and 3 specific clusters, respectively (Fig. 1). The phylogenetically remote species S. insectorum and S. pallida had the largest numbers of specific clusters (95 and 91, respectively; Fig. 1). Orthologous clusters that are specific to a certain species or in-paralog clusters may include groups of species-specific genes or lineage-specific gene expansion. Several genes remained ungrouped; the environmental species S. insectorum had the largest number of species-specific genes that do not belong to any orthologous cluster (Table 1). The lowest number of species-specific genes was found in S. brasiliensis (Table 1).

**TABLE 1:** Numbers of total genes and clusters and species-specific genes for *Sporothrix* spp.^a^

Species	Total no. of genes	No. of clusters	No. of species- specific genes
Sporothrix brasiliensis	9,091	8,525	395
Sporothrix schenckii	10,293	8,673	1,474
Sporothrix insectorum	9,496	7,768	1,380
Sporothrix pallida	9,712	8,167	1,200
Sporothrix globosa	8,747	8,479	176

a *In silico* gene prediction was performed by the use of AUGUSTUS (model S. schenckii) for S. pallida and *S. globosa*.

### Bottom-up proteomics.

Bottom-up analyses were done in S. brasiliensis using Sequest HT with Proteome Discoverer software v1.4.1.14 (Thermo Scientific); information about clinical type, geographic localization, and numbers of proteins for each strain is given in Table 2. The lowest number of proteins (594) was detected in strain ATCC MYA-4823, and the highest number (809) in the same species was detected in strain CBS 132992. Data corresponding to the molecular functions, biological processes, and cellular localizations for each protein are available (https://www.researchgate.net/publication/325224675_mSphere00514-17R2_TABLE_S1). No significant differences between strains were observed.

**TABLE 2:** Number of proteins detected for each strain used in bottom-up analysis^a^

Species	CBS identifier	Alternative identifier	Isolate location	Clinical type	No. of proteins
S. schenckii	132969	SSs80	Rio de Janeiro, Brazil	Human, skin biopsy specimen	664
S. schenckii	132978	Ss167	Peru	Soil	743
Sporothrix brasiliensis		ATCC MYA-4823	Rio de Janeiro, Brazil	Feline skin lesion	594
Sporothrix brasiliensis	132990	Ss54	Rio Grande do Sul, Brazil	Feline sporotrichosis	740
Sporothrix brasiliensis	132992	Ss82	Rio de Janeiro, Brazil	Human, secretion, arm injury	809

a The bottom-up analyses were done using Sequest HT with Proteome Discoverer software v1.4.1.14 (Thermo Scientific).

**FIG 2:**
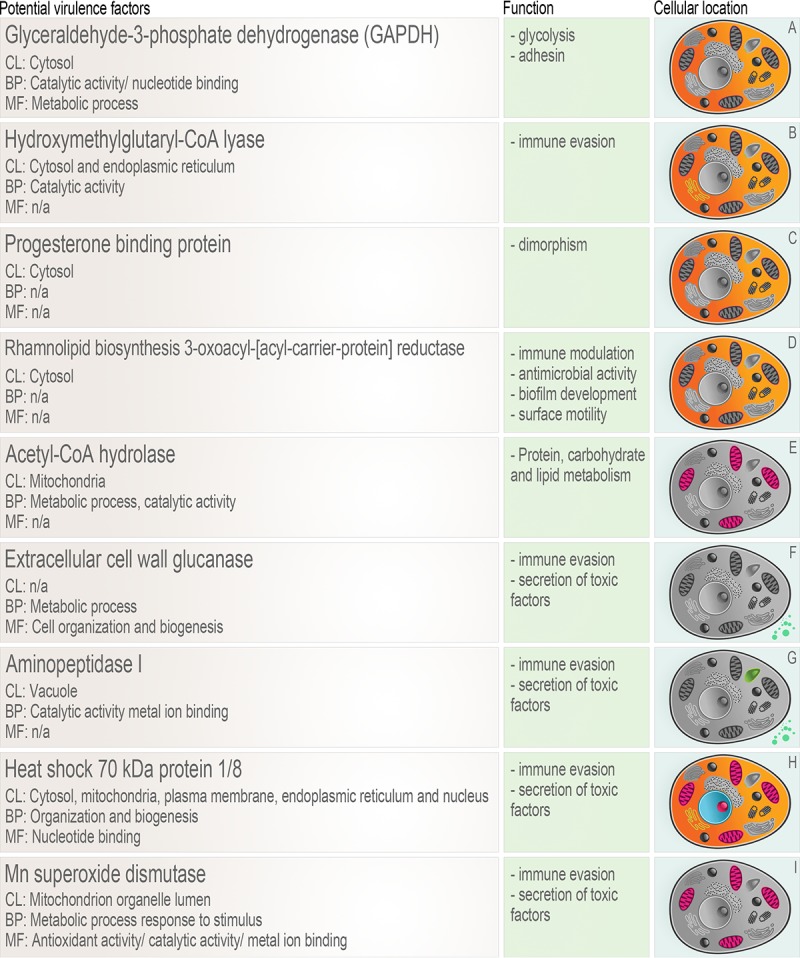
Proteins involved in virulence and immune evasion found differentially expressed in S. brasiliensis. CL, cellular localization; BP, biological process; MF, molecular function. The letters in the panels in the column at the right side of the figure represent references as follows: A, references 50, 51, 52, and 53; B, reference 54; C, reference 55; D, references 59, 60, and 61; E, references 57 and 58; F, reference 24; G, references 28, 29, and 39; H, references 47 and 48; I, references 40, 41, 42, and 43.

Comparing proteins common to S. brasiliensis strains to those analyzed and those of S. schenckii, we observed 541 and 355 proteins, respectively. Focusing on proteins exclusively expressed in each group, 60 unique proteins were found in S. brasiliensis and 87 in S. schenckii. All 60 unique proteins found in S. brasiliensis were present in the three strains of S. brasiliensis and absent in the two S. schenckii strains. A complete list of unique S. brasiliensis proteins, including descriptions, numbers of unique peptides, coverage, and peptide sequences, is available (https://www.researchgate.net/publication/325225115_Protein_Description_Unique_Peptides_Coverage_Peptides_Sequence). A literature search on the 60 proteins differentially expressed in S. brasiliensis revealed 9 proteins with potential impact on fungal virulence: extracellular cell wall glucanase, aminopeptidase I, Mn superoxide dismutase, heat shock 70-kDa protein 1/8, glyceraldehyde-3-phosphate dehydrogenase (GAPDH), hydroxymethylglutaryl-coenzyme A (HMG-CoA) lyase, progesterone binding protein, rhamnolipid biosynthesis 3-oxoacyl-(acyl-carrier-protein) reductase, and acetyl-CoA hydrolase. Information about functions and cellular locations in another fungal species is presented in Fig. 2.

### Genomics and proteomics.

Comparing the 60 proteins differentially expressed in S. brasiliensis against the groups of orthologs identified by OrthoMCL showed that all 60 proteins found in this species have a corresponding ortholog in S. schenckii, as expected from the genomic analysis, where no unique clusters were observed in S. brasiliensis. Seven proteins lacked orthologs in S. pallida and in S. globosa. These proteins are hypothetical protein SPBR_08110, hypothetical protein SPBR_05199, hypothetical protein SPBR_06610, hypothetical protein SPBR_08650, glutamate carboxypeptidase II, nucleic acid-binding protein, and Het-c. In S. globosa, nucleic acid-binding protein and Het-c were absent. We found paralogs of the hypothetical protein SPBR_08650 in four species studied, with the exception of S. globosa. In S. pallida, nucleic acid-binding protein and glutamate carboxypeptidase II were absent.

## DISCUSSION

Elucidation of the origin of virulence and characterization of the factors involved in the immune evasion are fundamental for understanding of microbe-host interactions. Pathogenic fungi have evolved efficient strategies to overcome killing by effector cells of the innate immune system. A widely accepted hypothesis to explain fungal virulence in opportunists is the continuous interaction of microbes with their environment and in competition with each other, resulting in the microbes acquiring survival strategies that lead to higher virulence when they accidentally find an animal host (36). However, in order to transmit the acquired properties to the next generations, they must be able to escape from the host, which is not possible for most opportunists (37). Therefore, distinction of free-living species from true pathogens, which are defined as transmissible fungi that, due to their specialized tissue phase, are able to escape from the host, is essential. Among their main traits, pathogens thus are recognizable by their specialized pathogenic phase. Recognizing potential virulence factors in this phase is fundamental to understand how the microorganisms escape from the immune system.

*Sporothrix* pathology has been puzzling because of the supposed opportunism of the fungi (the ancestral species S. schenckii occurring in plant material) combined with the presence of the invasive yeast form in mammalian tissue. The mycelium-to-yeast transition is a key developmental and ecological characteristic which is likely to be manifested in the protein profiles of the respective forms. *Sporothrix* species are relatives of insect-dispersed Ophiostomatales species (38), which lack a mammal-invasive yeast phase. In the “pathogenic clade,” S. schenckii and S. brasiliensis show low genetic divergence as established by using calmodulin as a barcoding marker (11). S. schenckii is associated with zoonoses and sapronoses, while the clonal offshoots S. brasiliensis and S. globosa are nearly exclusively zoonotic and sapronotic, respectively. In the present study, we analyzed differentially expressed proteins of the yeast form in S. brasiliensis and we focused on the proteins involved in virulence and evasion strategies against the immune system.

Our proteomic data were collected from the studied species under a specific condition, i.e., predominantly in the yeast form after a 4-day incubation at 35°C. Among the 60 proteins found exclusively expressed in S. brasiliensis, i.e., proteins with some modifications compared with their orthologues in S. schenckii, 9 had previously been implicated in virulence in other microorganisms. They have known roles in subversion of host innate immunity, dimorphic transition, extracellular vesicle production, aerobic respiration, immunogenicity, and invasiveness. The proteins concerned were cell wall glucanase, aminopeptidase I, Mn superoxide dismutase, heat shock 70-kDa protein 1/8, glyceraldehyde-3-phosphate dehydrogenase (GAPDH), hydroxymethylglutaryl-CoA lyase, progesterone binding protein, acetyl-CoA hydrolase, and rhamnolipid biosynthesis 3-oxoacyl-[acyl-carrier-protein] reductase.

Conserved orthologous genes corresponding to the 60 proteins found exclusively expressed in S. brasiliensis were found in S. schenckii. This suggests that determinations of modifications at the protein level are more informative than focusing on comparative genomics. The gene compositions of S. brasiliensis and S. schenckii are highly similar; S. brasiliensis does not possess species-specific clusters of orthologs.

One of the mechanisms used to cause damage to host cells is secretion of toxic factors via extracellular vesicles, as observed in Ascomycetes such as H. capsulatum, Candida albicans, C. parapsilosis, and S. schenckii, but this has also been observed in nonpathogenic Saccharomyces cerevisiae. A complex protein composition was revealed with multiple functions, including virulence factors (22–24). In S. brasiliensis, we identified several vesicle-associated proteins: extracellular cell wall glucanase, aminopeptidase I, Mn superoxide dismutase, and heat shock 70-kDa protein 1/8. The presence of extracellular vesicles is known in S. schenckii (23), and extracellular cell wall glucanase, one of the proteins transported by the vesicles, has now been found differentially expressed in S. brasiliensis. It induces macrophage lysis in Candida albicans, while components of the vesicles remodel the cell surface and induce host cell lyses in Cryptococcus neoformans. This suggests that it is a highly conserved mechanism among fungal pathogens (24).

Aminopeptidase I, a protein with a function in undermining mammalian innate host defense (29), was differentially expressed in S. brasiliensis. This protein may partially explain the high virulence of this species compared to other members of the pathogenic *Sporothrix* clade. In a Blastomyces dermatitidis murine model, the enzyme accounted for progressive pulmonary infection; elimination of expression led to attenuated virulence (28). Aminopeptidases are not exclusive to fungi but are also found in pathogenic bacteria and parasites (39).

Superoxide dismutases (SOD) contribute to growth and survival under conditions of oxidative stress, e.g., inside macrophages (40). We identified Mn superoxide dismutase in S. brasiliensis. The presence of SOD has been reported in S. schenckii (41), but the specific role of Mn SOD in virulence has yet to be addressed. In Cryptococcus gattii, virulence was reported to be dependent on Mn SOD, as mutants resulting from mutations of the Mn SOD gene presented attenuated virulence *in vivo* (42). A C. albicans Mn SOD mutant was more sensitive to stress than wild-type cells (43), confirming the importance of the protein for protection against the host defense.

Heat shock proteins (HSP) in fungi are produced in response to changes in temperature, enhancing pathogen survival in host tissue and promoting antifungal drug resistance (44), and are implicated in phase transition (45). We identified the 70-kDa heat shock protein differentially expressed in S. brasiliensis. This protein expressed on the surface of C. albicans cells mediated invasion of epithelial and endothelial cells *in vitro*, and the null mutant presented significantly attenuated virulence in murine models of oropharyngeal and disseminated candidiasis (46). In a murine model of meningoencephalitis caused by C. neoformans, the deletion of HSP 70 caused reduced laccase and melanin production, as well as attenuated virulence (47). The importance of HSP 70 in C. neoformans was confirmed with human alveolar epithelial cells, where the protein competed with glucuronoxylomannan, the major capsular antigen, in binding to host cells (48).

Adherence is essential to colonize mammalian host tissue. Glycoprotein-70 is an important cross-immunogenic adhesin that may undergo posttranslational modification to isoforms and glycoforms (34). In the present study, we identified in yeast the presence of another important adhesin, glyceraldehyde-3-phosphate dehydrogenase (GAPDH), involved in virulence and glycolysis (49). *In vitro* studies have shown that GAPDH-negative (GAPDH^−^) adhesin is capable of mediating entrance into the cell (50), while it was also shown to have an immunogenic role, with cells expressing this surface antigen both *in vitro* and in infected tissue (51). GAPDH of C. albicans is a fibronectin and laminin (52) and a plasminogen binding protein enhancing tissue invasion (53). In Paracoccidioides brasiliensis and H. capsulatum, GAPDH was present in larger amounts in the yeast phase than in the mycelium, suggesting a role in virulence (22).

Hydroxymethylglutaryl-CoA lyase is a metabolic enzyme that catalyzes the last step in leucine catabolism. The protein promoted survival and replication of H. capsulatum within the macrophage phagosome, as shown in a mouse model (54). HMG-CoA lyase deficiency results in accumulation of acidic compounds and in leucine metabolism impairment. The protein was found to be differentially expressed in S. brasiliensis.

Human infection by S. brasiliensis is more frequent in females than in males (2:1). This is associated with the relationship between cat owners (usually women/housewives) and their pets (14). However, a ligand for progesterone-adiponectin receptor was identified in S. schenckii (55), and here we identified a progesterone binding protein in S. brasiliensis which was not detected in any of the S. schenkii strains. The growth of dermatophytes was affected by progesterone hormones, and the effect of progesterone could be mediated through fungal intracellular receptors (56). This result should be investigated in order to confirm if the predominance in females is related to the pet/owner relationship or influenced by hormones.

Another protein differentially expressed in S. brasiliensis is acetyl coenzyme A hydrolase, a metabolite that has a central position in pathways for the utilization of glucose and other carbon sources. A relation with virulence was described for C. neoformans, where loss of acetyl coenzyme A (Acl1) resulted in a carbon source-dependent defect in the ability of the polysaccharide capsule to cause disease (57). Saccharomyces cerevisiae strains lacking acetyl-CoA hydrolase grow poorly on some alternative carbon sources and showed defects in pseudohyphal development (58), indicating a wider function of this protein.

Another significant protein in S. brasiliensis is rhamnolipid biosynthesis 3-oxoacyl-[acyl-carrier-protein] reductase, a surface-active glycolipid. Purified rhamnolipids act directly on immune cells. In Pseudomonas aeruginosa, they are involved in virulence, immune modulation, antimicrobial activity, biofilm development, and surface motility (59). Zulianello et al. (60) showed that P. aeruginosa requires rhamnolipids to invade respiratory epithelia reconstituted with primary human respiratory cells. Rhamnolipids also stimulate the release of mucus glycoconjugates from feline trachea or human bronchial mucosa (61).

The present paper is the first to characterize and compare the proteomes for the highly virulent species S. brasiliensis. The proteins described here are validated by literature comparisons where these targets have been reported as being significant for the virulence of other, highly diverse fungi. The mechanisms by which proteins are applied in virulence and immune escape have to be explored. Here, we describe a qualitative proteomics experiment in which we focused primarily on the identification. This is the first stage in the discovery of the virulence markers in S. brasiliensis, which need to be validated functionally in subsequent studies.

## MATERIALS AND METHODS

### Strains and culture conditions.

The analyzed S. schenckii strains (CBS 132969 and CBS 132978) and S. brasiliensis strains (CBS 132992, CBS 132990, and ATCC MYA-4823) were acquired from the reference collection of the Centraalbureau voor Schimmelcultures housed at Westerdijk Fungal Biodiversity Institute, Utrecht, The Netherlands. Yeast conversion was achieved by transferring mycelial colonies to brain heart infusion (BHI) liquid medium (Sigma-Aldrich) that was maintained at 35°C and shaken at 100 rpm for 7 days. Aliquots of liquid medium were transferred to BHI plates. For microscopy, cells were mounted on slides and viewed under an Olympus CH20 microscope.

### Protein extraction.

Converted yeast strains were cultivated for 4 days at 35°C. Yeast cells were collected, and 100 µl of lysis solvent comprised of acetonitrile (ACN) and formic acid was added. Sonication (probe sonicator; Thermo Fisher Scientific) was applied for 1 min to lyse the cells and release the proteins, followed by centrifugation (2,000 × *g*, 2 min) to pellet the cell debris. The supernatant was removed, and 100 µl of ACN and formic acid was added. The supernatant was stored at −80°C. For all the strains, we used three biological replicates and three technical replicates.

### Sample preparation.

Extracts were dried (Concentrator Plus; Eppendorf, Hamburg, Germany) for 2 h and reconstituted in 100 µl of ammonium bicarbonate (ABC; 50 mM) with the aid of sonication (Branson 1-291; Branson, Danbury, CT) for 10 min. To reduce disulfide bonds, 2.4 µl of DTT (1,4-dithiothreitol; Sigma-Aldrich) (10 mM) was added followed by incubation at 60°C for 45 min. Alkylation was performed using 5 µl of 30 mM iodoacetamide (Sigma-Aldrich) and incubation at room temperature in the dark for 45 min. Subsequently, 10 µl of trypsin (Sigma-Aldrich) diluted in ammonium bicarbonate (ABC) (100 ng/µl) was added. Samples were maintained at 37°C overnight, and then 2 µl of formic acid (Optima; Fisher Chemical, Wilmington, DE, USA) (liquid chromatography/mass spectrometry [LC/MS] grade) was added, followed by incubation of the samples at 37°C for 10 min. After centrifugation (1,400 rpm, 5 min), the supernatant was transferred to another tube and dried for 2 h (Concentrator Plus; Eppendorf, Hamburg, Germany). A 200-µl volume of 0.1% formic acid (Optima, Solvent Blends; Fisher Chemical) (LC/MS grade)–water was used to reconstitute the samples for analysis. As a control, HeLa Protein Digest Standard (Pierce–Thermo-Fisher) was used.

### Mass spectrometry.

Tryptic digests (peptides) were analyzed by online nanoflow LC using an Easy-nLC II system (Thermo Fisher Scientific). Peptides (5-µl injection) were separated with a 60-min gradient (0 to 35% buffer B over 50 min, 35 to 80% buffer B over 5 min [held at 80% buffer B for remaining 5 min]) (buffer A, 0.1% formic acid–2% acetonitrile; buffer B, 0.1% formic acid–0.1% acetonitrile) at a flow rate of 200 nl/min (45°C) on a EasySpray column (PepMap RSLC ES802; Thermo Fisher Scientific) (75 µm by 25 cm). HeLa Protein Digest Standard (Pierce–Thermo-Fisher) was used as a control. The Easy-nLC system was coupled to a QExactive-HF mass spectrometer via an Easy-Spray electrospray ion source (Thermo Fisher Scientific). All samples were measured in a data-dependent acquisition mode using a scan range of 350 to 2,000 *m*/*z* (MS1) in the Orbitrap, 5 microscans, an automatic gain control (AGC) setting of 3 × 10^6^, and 120-k resolution. Fragmentation was performed in a data-dependent manner with a normalized collision energy value of 27 for the top 5 peaks in each MS scan (MS2 isolation window of 2.0 *m*/*z*, 60-k resolution, 1 microscan, and an AGC setting of 1 × 10^5^ with a maximum ion injection time of 60 ms and a dynamic exclusion time of 60 s). MS measurements were randomized to eliminate technical artifacts.

Protein identification was carried out with mass spectrum raw files using Sequest HT with Proteome Discoverer software version 1.4.1.14 (Thermo Fisher Scientific). Searches were made against a Sordariomycetes database, made up of all protein sequences for the taxonomy downloaded from Uniprot-Swiss-Prot (http://www.uniprot.org) to identify peptides and proteins. We searched high-confidence peptides, including at least one unique peptide per protein. Protein Center (Thermo Fisher Scientific) was used for data analysis and for generating plots. Gene ontology (GO) terms at Candida Genome Database (CGD) were used to classify proteins.

### Genomics.

The protein sequences of S. brasiliensis (GCA_000820605), S. schenckii (GCA_000961545), and Sporothrix insectorum (GCA_001636815) were retrieved from GenBank. Additionally, the protein set of S. schenckii was used for training AUGUSTUS (62) for *ab initio* prediction of genes in the genomes of S. pallida and S. globosa using default parameters. The proteins corresponding to these five *Sporothrix* spp. was assessed for orthologous and paralogous identification using OrthoVenn (35). The Orthomcl (63) parameters were set to a Markov inflation value of 1.5 and a maximum E value of 10^−5^. Species-specific proteins were extracted using a custom Bash script.

### Accession number(s).

Regarding the proteomic data, the analyses were performed using sequences deposited in GenBank. The GenBank accession numbers are as follows: extracellular cell wall glucanase (KIH90339.1), aminopeptidase I (KIH92463.1), Mn superoxide dismutase (XP_014168450.1), heat shock 70-kDa protein 1/8 (KIH91446.1), glyceraldehyde 3-phosphate dehydrogenase (GAPDH) (KIH93501.1), hydroxymethylglutaryl-CoA lyase (KIH90753.1), progesterone binding protein (KIH94433.1), rhamnolipid biosiosynthesis 3-oxacyl-(acyl-carrier-protein) reductase (SPBR_07600), acetyl-CoA hydrolase (KIH86467.1), and NADH ubiquinone oxidoreductase subunit B17.2 (KIH87935.1).
